# 529 Outcomes of Total Parenteral Nutrition Use in Burn Patients at a Single Institution

**DOI:** 10.1093/jbcr/irac012.159

**Published:** 2022-03-23

**Authors:** Sanja Sljivic, Lori Chrisco, Cindy A Long, Brian P McKinzie, An Bui, Rabia Nizamani, Booker King, Felicia Williams

**Affiliations:** University of North Carolina - Chapel Hill, Chapel Hill, North Carolina; North Carolina Jaycee Burn Center, Chapel Hill, North Carolina; UNC Medical Center, Chapel Hill, North Carolina; UNC, Chapel Hill, North Carolina; Campbell School of Osteopathic Medicine, Fayetteville, North Carolina; UNC Jaycee Burn Center, Chapel Hill, North Carolina; UNC Jaycee Burn Center, Chapel Hill, North Carolina; UNC Jaycee Burn Center, Chapel Hill, North Carolina

## Abstract

**Introduction:**

Total parenteral nutrition (TPN) has been widely used among critically ill patients. Some of the controversy surrounding parenteral nutrition stems from its early use in the 1980s, which primarily focused on hyperalimentation. In burn patients, nutritional support is a critical aspect of treatment. The metabolic rate in this patient population can be greater than twice the normal rate, and this hypermetabolic response can last more than a year after the burn injury has occurred. The objective of this study was to evaluate the outcomes of patients in our Burn Center who received TPN during their hospitalization.

**Methods:**

This was a single-site, retrospective review using our institutional Burn Center registry. All adult patients (18 years or older) admitted to our Burn Center between July 1, 2015 and June 30, 2021 who had received TPN during their hospitalization were included in this study. Adult patients who had not received TPN were included for comparative purposes. Variables of interest included demographics, burn mechanism, length of stay (LOS), ICU and ventilator days, and mortality.

**Results:**

There were 20 burn patients who received TPN during their hospitalization. Of those patients who received TPN, 90% were male. The mean age was 45 years, and the mean total body surface area (TBSA) involvement was 32%. The mean resting energy expenditure (REE) was 3,084. On average, the time from day of admission to initiation of TPN was 40 days, and the mean length of TPN administration was 20 days. The overall decrease in patient weight from admission to discharge was 10%. The mean LOS for the TPN group was 118 days. The mean LOS in the ICU was 92 days. The mean ventilator days were 89 days. The overall hospital mortality of patients who received TPN was 20%. When matched with patients who had similar TBSA involvement and who had not received TPN, there was no difference in mortality. However, there was a significant difference in weight loss (4% for non-TPN group), overall LOS (63 days), ICU LOS (29 days), and ventilator days (31 days).

**Conclusions:**

Burn patients who received TPN during their hospitalization had a greater decrease in their overall weight, had a longer hospital and ICU length of stay, and were ventilated longer than those patients who did not receive TPN. These findings are to be expected given that patients who receive TPN tend to be more critically ill, and therefore, require more nutritional support.

